# A Successful Endodontic Outcome with Non-Obturated Canals

**DOI:** 10.7508/iej.2015.03.013

**Published:** 2015-07-01

**Authors:** Saeed Asgary, Mahta Fazlyab

**Affiliations:** a * Iranian Center for Endodontic Research, Research Institute of Dental Sciences, Dental School; Shahid Beheshti University of Medical Sciences, Tehran, Iran*

**Keywords:** Biomaterials, Calcium-Enriched Mixture, CEM Cement, Coronal Seal, Endodontics, SealBio, Root-End Surgery

## Abstract

This case report represents the outcome of endodontic treatment in an infected mandibular molar with periradicular periodontitis and inherent poor prognosis of root canal treatment due to severe root curvature. The tooth was successfully treated by leaving the mesial root non-obturated, the canal orifices were coronally sealed with calcium enriched mixture cement and a definitive coronal amalgam restoration, was placed at the subsequent visit.

## Introduction

In 1967, Grossman described the principles of root canal therapy (RCT) [1]; the 9^th^ principle was termed *hermetic seal* of the canal meaning airtight seal which was later replaced with *impermeable* seal. Schilder [2] described the aim of endodontic treatment as *total obturation* of the root canal space, that prevents the bacterial migration from endodontium to periodontium. Ten years later this concept was challenged by Dubrow [3] who reported a case that was healed after instrumentation, medication and perfect coronal sealling without obturation of the canals. 

The importance of *coronal* seal has been recognized in dental literature; sealed root canals can be coronally recontaminated if a recurrent decay exposes the endodontic filling material [4]. When this situation occurs, the coronal portion of the root canal is exposed to the oral flora, which can allow egress of bacteria to the periapical tissues [4]. 

Today the ultimate goal of endodontic treatment has stepped forward; even with conventional gutta-percha obturation, the ultimate aim is to achieve a cemental/fibrous barrier at the root apex [5]. In theory, if we can sterilize the canal and prevent coronal leakage, then a root filling should not be necessary. Shah and Logani [6] reported that placement of calcium-sulphate cement over the orifices of none-obturated cleaned/shaped canals and permanent restoration, or in their term *SealBio*, was successful after short- and long-term evaluation.

This report represents the treatment outcome of an infected tooth with inherent poor prognosis of endodontic treatment that was successfully treated with the same concept: coronal sealing without root obturation.

## Case Report

**Figure 1:**
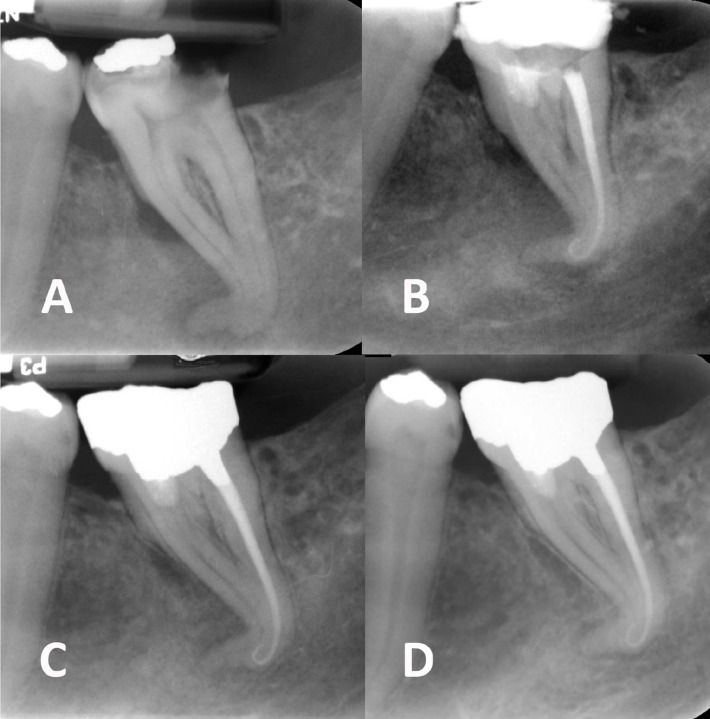
*A) *Pre-operative periapical radiography;* B) *Post-operative image; *C-D) *Short- and long-term (12-month) follow-up images

A 27-year old male complaining of a dull pain in his lower left jaw referred to a private dental clinic. On clinical inspection, no swelling was detected on his face. Intra-oral evaluation showed a severely decayed second mandibular molar that was mesially tilted and had an occlusal amalgam filling; the tooth was strategic, as its absence would lead to free-end edentulism. The tooth was tender on palpation but was not responsive to cold and electric pulp testing. On periapical radiography fading of the lamina dura and periradicular bone trabeculation was evident around the apex of the severely curved roots and in the middle area surrounding the mesial root (Figure 1A); periradicular periodontitis subsequent to pulpal necrosis was the final diagnosis. 

The treatment option was discussed with the patient; RCT encompassing the high possibility of instrument fracture due to the severely curved roots. He was informed that in case of instrument separation, periapical surgery should be planned which was also unpredictable because of the long roots, proximity of root apices with mandibular canal and low depth of the vestibule. He chose coronal sealing so that in case of failure, other low-prognosis options would be tried.

After local anesthesia, all caries were removed and an access cavity was prepared. Then the tooth was isolated and the canal orifices were located. The distal canal was navigated with a #10 hand NiTi K-file (Maillefer, Ballaigues, Switzerland) and its working length (WL) was determined with an electronic apex locator (Root ZX, J. Morita USA, Inc., Irvine, CA, USA). Although the mesial canals were navigated to the similar WL as the distal canal, due to the relatively higher root curvature in the apical area and also the obtuse curvature of the mesial canals branching from the chamber floor, the largest file that could penetrate the mesial canals was #25. Even troughing of the mesial part of the chamber floor did not facilitate negotiating the canals; the anatomy condemned these canals untreatable. All canals were irrigated with 5.25% NaOCl solution and the apical part of the distal canal was prepared to #25 with inter-instrumentation irrigation. For disinfection, canals were filled with a creamy paste consisting of minocycline (Razak, Tehran, Iran), ciprofloxacin (Amindaru, Tehran, Iran) and metronidazole (Parsdaru, Tehran, Iran) in equal proportions mixed with saline. 

Two weeks later the patient was asymptomatic. The canals were irrigated, cleaned and dried; distal canal was obturated with #25/0.04 master gutta-percha cone and lateral condensation technique using Roth 801 root canal sealer (Roth international LTD, IL, USA). For the mesial canals, calcium-enriched mixture (CEM) cement (BioniqueDent, Tehran, Iran) was mixed according to the manufacturer’s instructions and then placed in the coronal part of the mesial canals. The tooth was temporarily restored with Cavit (ESPE-Premier, Norristown, PA, USA) which was later replaced with amalgam permanent restoration (Figure 1B-C). 

During the next 12 months, the tooth was asymptomatic and functional; moreover, radiography displayed the reestablishment of the periodontal ligament and lamina dura (Figure 1D). 

## Discussion

This report discussed the successful treatment outcome of a necrotic tooth with apical periodontitis by sealing the mesial canal orifices with CEM cement. 

The concept of reported treatment is not “the trailblazer” in Endodontics. It is a proved fact that the fundamental of endodontics is based on removing the cause of infection /inflammation and preventing its reoccurrence by means of sealing [7-9]. In 1965 the classic study by Kakehashi *et al.* [10] showed the role of bacteria in establishment of periapical infection. In 1992, Gutman *et al.* [9] put an emphasis on the importance of elimination of bacteria and prevention of their reentrance. Endodontically treated teeth fail not because of poor filling but due to poor cleaning and shaping [11]; besides, the maintenance of healthy periapical area largely depends on the quality of coronal seal rather than that of root filling [12]. In an interesting study, Sabeti *et al.* [11] evaluated the role of obturation in periapical repair in dogs’ teeth, through creating apical pathosis by leaving the tooth open for 6 weeks; then the canals were cleaned and shaped. They sealed the orifices of the nonobturated canals with bonding and amalgam. They reported that there was no statistically significant difference between the obturated and non-obturated teeth regarding bone resorption, inflammation, thickness of periodontal ligament and in one term healing of the periapical lesion [11]. 

The success of treatment is known to be dependent on the integrity of proper cleaning and shaping and chemical irrigation [13]. There is an interesting report of 18 teeth with periapical infection that after cleaning and shaping and irrigation with 2.5% NaOCl, where medicated with triple antibiotic paste; the session after, the canal orifices of all teeth were sealed with calcium-sulphate based cement. After 6 to 36 months of follow-up, all symptoms of all cases had resolved [6]. Authors named this technique as “SealBio”. Therefore, it can be concluded that in selected cases that leave the clinician with no other choices, the same as the present case, antibiotic medication as well as profound sealing of the canals give space to successful outcome.

The canal disinfection protocol in this treatment included the antibiotic paste. The most commonly used antibiotic for this purpose is the equal mixture of metronidazole, minocycline, and ciprofloxacin, called the triple antibiotic paste [14], first introduced by Hoshino [15]. The protocol of root canal disinfection using triple antibiotic paste in regenerative endodontic treatment was first attempted by Banchs and Trope [16]. Since then, several clinical studies have been published showing that this method of disinfection is being clinically and radiographically successful [14, 17] because it can penetrate deep layers of dentin [15].

In the present report, CEM cement was used to seal off the non-obturated mesial canals. CEM cement setting expansion is almost similar to mineral trioxide aggregate (MTA) that alongside with reasonable flow and film thickness [18], enables the cement to effectively seal the area and prevent microleakage [19]. The sealing ability and biocompatibility of CEM cement is favorable and similar to MTA [20-23]. The small particle size of CEM in comparison with MTA can also justify its favorable sealing properties [18]. Apart from sealing, the success of treatment can be attributed to antimicrobial properties of this bioceramic due to its alkaline pH (~11). Antibacterial effects of CEM cement is similar to calcium hydroxide and superior to MTA [24] and its lethal effect on *Candida albicans *is similar to MTA after 24 h [25]. Although the outcome of the present case supports the usage of CEM in similar cases, such reports own the lowest level of evidence in the evidence-based practice. 

## Conclusion

The reported modified endodontic treatment for a necrotic/symptomatic mandibular molar is based on clinical rational. Further trials with longer follow-up and larger sample sizes are needed to evaluate the outcome of such treatments.
